# Development of acute lung injury or acute respiratory distress syndrome after subarachnoid hemorrhage, predictive factors, and impact on prognosis

**DOI:** 10.1007/s13760-023-02207-z

**Published:** 2023-03-15

**Authors:** Jiang Wu, Wei Gao, Hongrong Zhang

**Affiliations:** Department of Neurosurgery, The First Affiliated Hospital of Soochow University, 899 Pinghai Road, Suzhou, 215026 Jiangsu People’s Republic of China

**Keywords:** Acute lung injury, Acute respiratory distress syndrome, Aneurysmal subarachnoid hemorrhage, Risk factors, Prognosis

## Abstract

Acute lung injury or acute respiratory distress syndrome (ALI/ARDS) is a common complication after aneurysmal subarachnoid hemorrhage (aSAH), and is associated with worse neurologic outcomes and longer hospitalization. However, the effect of ALI/ARDS in SAH has not been well elucidated. The purpose of this study was to determine the incidence of ALI/ARDS in a cohort of patients with SAH and to determine the risk factors for ALI/ARDS and their impact on patient prognosis. We performed a retrospective analysis of 167 consecutive patients with aSAH enrolled. ALI/ARDS patients were rigorously adjudicated using North American-European Consensus Conference definition. Regression analyses were used to test the risk factors for ALI/ARDS in patients with SAH. A total of 167 patients fulfilled the inclusion criteria, and 27% patients (45 of 167) developed ALI. Among all 45 ALI patients, 33 (20%, 33 of 167) patients met criteria for ARDS. On multivariate analysis, elderly patients, lower glasgow coma scale (GCS), higher Hunt-Hess grade, higher simplified acute physiology score (SAPS) II score, pre-existing pneumonia, gastric aspiration, hypoxemia, and tachypnea were the strongest risk factor for ALI/ARDS. Patients with ALI/ARDS showed worse clinical outcomes measured at 30 days. Development of ALI/ARDS was associated with a statistically significant increasing the odds of tracheostomy and hospital complications, and increasing duration of mechanical ventilation, intensive care unit (ICU) length and hospitalization stay. Development of ALI/ARDS is a severe complication of SAH and is associated with a poor clinical outcome, and further studies should focus on both prevention and management strategies specific to SAH-associated ALI/ARDS.

## Introduction

Aneurysmal subarachnoid hemorrhage (aSAH) is one of the common subtypes of hemorrhagic stroke and critical illnesses in neurosurgery, with a high rate of death and disability. The mortality rate due to aSAH is between 35 and 40%, and the disability rate among aSAH survivors reaches 50% [1]. This overall poor prognosis is influenced by initial hemorrhage and several others complications such as rebleeding and delayed cerebral ischemia and medical complications. Therefore, non-neurologic complications may also be responsible for poor outcomes after SAH [2]. Pulmonary complications are particularly frequent after SAH [3]. Acute lung injury or acute respiratory distress syndrome (ALI/ARDS), the most severe form of lung injury and was the most frequent non-neurologic severe organ system failure [4], it is lead to the severity of the respiratory failure. Persistent hypoxemia was secondary to nerve cell damage and forms a vicious cycle, which leads to the complication of clinical management and seriously affects patient prognosis.

Previous studies have reported that 4–18% of patients with SAH also suffered ALI/ARDS [2, 5–7], whereas the molecular mechanism underlying it remains largely speculative. Preliminary clinical studies have suggested that mechanisms of ALI/ARDS-associated acute brain injury (ABI, including acute ischemic stroke, SAH, cerebral hemorrhage, and traumatic brain injury) include primary hypoxic-ischemic injury from hypoxic respiratory failure and secondary injury from lung injury-induced neuroinflammation and increased intracranial pressure (ICP) from lung-protective mechanical ventilatory strategies, and adverse side effects of therapeutic management. etc. [8, 9]. Although trials and clinical guidelines are available to guide and support the primary treatment of SAH and ALI/ARDS respectively, the therapeutic options of ALI/ARDS in SAH patients remain limited, and even sometimes the treatment strategies for SAH may conflict with the management of ALI/ARDS [10]. Therefore, early prevention and diagnosis of ALI/ARDS and appropriate treatment measures to maximize patient prognosis are crucial in clinical work. The primary aim of this investigation was to determine potential risk factors for ALI/ARDS, clinical outcomes, and to hopefully provide updated epidemiological data on ALI/ARDS after SAH.

## Methods

### Study patients

The study was conducted at the First Affiliated Hospital of Soochow University. All procedures were approved by the Clinical Studies Ethic Committee at The First Affiliated Hospital of Soochow University (Suzhou, China; approval no. 2022–087). We conducted a retrospective cohort analysis of 167 patients with SAH. All patients with aSAH admitted between Jan 1, 2018, and Dec 31, 2021, were eligible for the study. Patient characteristics are recorded in detail and accurately, including age, gender, smoking habits, history of surgery and trauma, medication, history of hypertension, coronary heart disease, lung disease and other chronic diseases. All patients are supervised by neurocritical care specialist with extensive clinical experience.

Inclusion criteria: Age ≥ 18 years; SAH diagnosed clinically and confirmed by a computed tomography (CT) scan and cerebral artery aneurysm was confirmed by the means of angiography; Presence of information regarding the outcome at intensive care unit (ICU) or hospital discharge. Exclusion criteria: Age < 18 years; Non-aneurysmal SAH; Patients who died within 48 h of admission; No available data on the outcome; ALI/ARDS diagnosed within 24 h of admission.

The neurologic condition at the time of admission was scored by the neurosurgeon according to the classification of Hunt-Hess, Glasgow Coma Scale (GCS) [11]. The Simplified Acute Physiology Score (SAPS) II and modified Fisher scale (mFisher) were used to measure the severity of SAH [12, 13]. SAH diagnosed clinically and confirmed by a CT scan and a cerebral artery aneurysm was confirmed by the means of angiography. ALI/ARDS patients were diagnosed and registered on the day of onset. Including PaO_2_/FIO_2_ (P/F) ratios and the presence of bilateral infiltrates which were reviewed independently by two blinded radiologists were recorded in detail. All patients were treated, cared for, and registered daily by trained neurocritical care physicians and nurses. ALI/ARDS was defined according to the North American-European Consensus Conference definition of acute arterial hypoxemia (P/F ≤ 300 mmHg, P/F ratios was confirmed in three consecutive blood gases), bilateral infiltrates on chest radiograph, and pulmonary artery wedge pressure ≤ 18 mmHg or no clinical evidence of left atrial hypertension [14]. ARDS was defined as ALI with a ratio of P/F ≤ 200 mmHg. And note whether an extraventricular drain (EVD) is performed to monitor the patient's invasive ICP. Patients were discharged or discharged from the ICU with neurological outcomes assessed by the Glasgow Outcome Score (GOS) [15, 16]. We recorded the length of mechanical ventilation and ICU length of stay in days, tracheostomy, and hospital complications.

Pre-onset mechanical ventilation tidal volume [(calculate the predicted body weight (PBW) based on height, then calculate the tidal volume per kilogram of body weight and set > 8 ml/kg as high tidal volume)], GCS, aspiration and vomiting, vital signs (including respiratory rate, heart rate, blood pressure, oxygen saturation) were collected. Hypoxemia on hospital presentation was defined as initial oxygen saturation < 95%, and tachypnea was defined as respiratory rate > 30 breaths/min. Patients with aSAH who did not develop ALI/ARDS during the same period were served as controls. Patients with aSAH complicated by ALI/ARDS were taken from the ALI/ARDS registry as described previously. For those patients who received ICP monitoring, the ICP was measured continuously and recorded by the nurse regularly. All patients with ALI/ARDS are treated with lung-protective ventilation, all ventilator settings were transcribed from respiratory therapy flow sheets. For patients meeting the criteria for ALI/ARDS, ventilation variables and arterial blood gas measurements were collected on days 1, 3, and 7 of ALI/ARDS. Data were collected for 30 days following admission. Vasospasm was measured by screening transcranial doppler examinations.

## Statistical analysis

Values are expressed as mean ± SD or frequency (percent). Bivariate analysis of continuous variables was performed using an unpaired Student’s *t*-test for normally distributed variables or the Wilcoxon rank-sum test for nonnormally distributed variables. We compared categorical variables using the chi-square test or Fisher’s exact test, as appropriate. Multivariate stepwise regression analysis was performed to identify the risk factors. All analyses were conducted using SPSS 25.0 software (IBM Corp.), and *p* < 0.05 was considered significant.

## Results

### Patients with SAH characterized according to the presence of ALI/ARDS

A total of 167 patients with aSAH were admitted during the study period and survived > 48 h. In those patients, 27% (45 of 167) developed ALI. Table 1 shows the main characteristics of our cohort. Most patients develop ALI/ARDS within 5 days. Among all ALI patients, 33 (20%, 33 of 167) patients met the criteria for ARDS. Groups were similar in terms of gender, vasospasm, pre-onset ventilation strategy, and percent receiving operative management. However, patients with ALI/ARDS tended to have higher severity of SAPS II scores, worse Hunt-Hess grade, worse mFisher scale, and a lower GCS on admission. ALI/ARDS patients also tended to have a higher maximum ICP during their hospitalization. Moreover, patients elder than 60 years are more likely to develop ALI/ARDS. Patients with prior aspiration or aspiration pneumonia are more likely to develop ALI/ARDS. Similarly, patients with ALI/ARDS have higher systolic blood pressure, respiratory rate, and lower oxygen saturation before the onset of the disease. We also examined pre-existing underlying diseases and found that in patients who developed ALI/ARDS, there was a higher prevalence of the pre-existing respiratory disease. However, patients of smoked or vascular comorbidities did not show a higher incidence of ALI/ARDS.

**Table 1:** Characteristics of study cohort stratified by development of ALI/ARDS

	NO ALI/ARDS	ALI/ARDS	*p* Value
Patients (*N*, %)	122 (73.1%)	45 (26.9%)	
Age, years ± SD	62 ± 13	69 ± 12	0.001
≥ 60 (*N*, %)	55 (45.1%)	31 (68.9%)	0.006
< 60 (*N*, %)	67 (54.9%)	14 (31.1%)	
Male sex (*N*, %)	56 (45.9%)	17 (37.8%)	> 0.05
Hunt-Hess grade ± SD	2.6 ± 1.1	3.4 ± 1.2	< 0.001
SAPS II score ± SD	27 ± 12	39 ± 14	< 0.001
Modified Fisher scale ± SD	2.2 ± 1.3	3.3 ± 1.4	< 0.001
Admission GCS (*N*, %)			
3–5	13 (10.6%)	11 (24.4%)	0.024
6–13	64 (52.5%)	26 (57.8%)	
14–15	45 (36.9%)	8 (17.9%)	
Persistent maximum systolic pressure on admission, mmHg ± SD	142 ± 69	167 ± 57	0.018
Primary management			
Endovascular (*N*, %)	39 (31.9%)	12 (26.7%)	> 0.05
Operative (*N*, %)	83 (68.1%)	33 (73.3%)	
Gastric aspiration or pneumonia (*N*, %)	21 (17.2%)	17 (37.8%)	0.005
Mechanical ventilation (*N*, %)	49 (40.2%)	45 (100%)	
Duration of mechanical ventilation in days ± SD	4.3 ± 3.6	12.6 ± 8.7	< 0.001
Percent of time high tidal volume ventilation before the onset of ALI^A^ (% ± SD)	67 ± 34	71 ± 31	> 0.05
Hypoxemia before the onset of ALI (*N*, %)	12 (9.8%)	11 (24.4%)	0.015
Tachypnea (*N*, %)	7 (5.7%)	8 (17.8%)	0.028
EVD insertion (*N*, %)	69 (56.6%)	37 (82.2%)	
Maximal ICP^B^, mm Hg ± SD	13 ± 8	21 ± 15	0.001
Vasospasm (*N*, %)			
None	38 (31.1%)	12 (26.7%)	> 0.05
Mild-moderate	67 (54.9%)	26 (57.8%)	
Severe	17 (13.9%)	7 (15.5%)	
Patient Comorbidity on admission, (*N*, %)			
Respiratory Disease^C^	19 (15.6%)	14 (31.1%)	0.025
Smoking	15 (12.3%)	7 (15.5%)	
Vascular Comorbidities^D^	54 (44.3%)	21 (46.7%)	
ALI/ARDS onset day ± SD	NA	4.1 ± 1.5	

^A^Mechanical ventilation tidal volume [(calculate the predicted body weight (PBW) based on height, and set > 8 ml/kg as high tidal volume)]

^B^Highest sustained ICP observed throughout the hospitalization

^C^Respiratory disease defined as severe chronic lung disease, chronic asthma, cystic fibrosis, or chronic obstructive pulmonary disease (COPD)

^D^Vascular comorbidities defined as hypertension, myocardial infarction, diabetes mellitus, cerebralvascular accident, cardiovascular disease, coronary stenosis and angina

### Factors associated with ALI/ARDS following SAH

Table 2 describes factors associated with the development of ALI/ARDS. Increasing age, lower GCS, higher Hunt-Hess grade, and higher SAPS II score was associated with increased odds of developing ALI/ARDS. Admission, development of pneumonia, gastric aspiration, hypoxemia, and tachypnea were associated with an increased incidence of developing ALI/ARDS.

**Table 2:** Multivariate analysis for factors associated with the development of ALI/ARDS in patients with subarachnoid hemorrhage

Factors	OR	95% CI	*p* Value
Age ≥ 60	2.68	1.31–5.49	0.007
GCS	0.94	0.91–0.97	0.003
Hunt-Hess grade	1.32	1.09–1.60	0.026
SAPS II	1.24	1.08–1.43	0.032
Gastric aspiration or pneumonia	2.92	1.36–6.27	0.006
Hypoxemia at presentation	2.96	1.20–7.41	0.018
RR (respiratory rate)	3.55	1.21–10.51	0.021

Respiratory rate and *oxygen saturation* were collected daily and averaged from three values. Aspiration of gastric contents were only counted if they occurred prior to the onset of ALI/ARDS

### Association between ALI/ARDS and patients’ outcome

Table 3 describes the prognosis of patients associated with the development of ALI/ARDS. Patients with ALI/ARDS showed worse GOS compared to those who did not develop ALI/ARDS. The development of ALI/ARDS was associated with increased odds of tracheostomy and hospital complications. Overall ventilator parameters did not differ significantly between ventilation days meeting ALI/ARDS criteria and those that did not. Patients who developed ALI/ARDS had a longer duration of mechanical ventilation, ICU length and hospitalization of stay.

**Table 3:** Association between ALI/ARDS and patients’ outcome

	NO ALI/ARDS	ALI/ARDS	*p* Value
Patients (*N*, %)	122 (73.1%)	45 (26.9%)	
GOS at 30 day (*N*, %)			
Good/moderate	79 (64.8%)	21 (46.7%)	0.034
Vegetative/severe	43 (35.2%)	24 (53.3%)	
Dead	0	0	
Tracheostomy (*N*, %)	15 (12.3%)	12 (26.7%)	0.025
Ventilation time in days ± SD	4.3 ± 3.6	12.6 ± 8.7	< 0.001
Duration of ICU in days ± SD	8.2 ± 3.5	16.7 ± 11.4	< 0.001
Length of hospitalization in days ± SD	19.2 ± 14.6	27.3 ± 20.1	0.013
Hospital complications^A^ (*N*, %)	57 (46.7%)	32 (71.1%)	0.005

^A^Hospital complications defined as status epileptic, infectious diseases, cardiac dysfunction, congestive heart failure, hypertension, metabolic dysfunction, neurological

## Discussion

ALI/ARDS is a common in-hospital complication after admission for SAH. In our study, the prevalence of ALI and ARDS was 27% and 20%, respectively, and this is consistent with the rate of 20–25% observed in studies of ALI/ARDS development after SAH [5, 7, 17, 18]. The early onset of ALI/ARDS in patients with SAH has been mentioned in many studies. The insidious onset and rapid progression make early diagnosis and treatment extremely difficult. Currently, little is known about the etiology of ALI/ARDS in SAH. Several mechanisms have been postulated, including aspiration, infection, and neurogenic pulmonary edema [19]. The occurrence of ALI/ARDS not only provides a susceptible environment for pulmonary infections commonly seen after SAH but also directly causes diffusion dysfunction of intrapulmonary oxygen, resulting in hypoxemia that further aggravating secondary damage to the nervous system, which has become one of the most important complications affecting the patients' prognosis and leading to death. The results of this study showed that the incidence of ALI/ARDS was significantly higher in patients with advanced age, clinical grade of hemorrhage, aspiration, and low GCS scores.

It has reported that high tidal volumes (> 8 ml/kg, estimated body weight) are associated with ALI/ARDS development during mechanical ventilation [17, 20, 21]. It is possible that the higher tidal volume produces volume-related lung injury, increased pulmonary vascular permeability, and decreased alveolar surfactant, which contribute to the development of ALI/ARDS. We reviewed the ventilator settings parameters of patients before the onset of ALI/ARDS. Interestingly, unlike in previous studies, although in ALI/ARDS patients, the duration of high tidal volume ventilation is relatively long, high tidal volume ventilation is not a risk factor for the development of ALI/ARDS in our cohort. It is possible that our sample size was too small to allow the detection of an independent association between ventilator parameters and the development of ALI/ARDS. Studies have demonstrated that a mechanical ventilation strategy using low tidal volumes and reduced end-expiratory pressures reduces mortality and increase ventilator-free days in patients with ALI [22]. It should be noted, however, that small tidal volume ventilation may lead to increased ICP and cerebral hypoxia. Invasive ICP and real-time monitoring of brain tissue oxygen may be performed. If direct measurement is not feasible, control of ventilation (6–8 ml/kg) should be done as much as possible to maintain PaCO_2_ at normal levels (35–45 mmHg) from the perspective of lung compliance [23]. In addition, previous studies have reported that the recommended PaO_2_ target for brain-injured patients with healthy lungs is above 75 mmHg, while lower PaO_2_ targets (55–80 mmHg) are recommended for patients without brain injury [24, 25].

Brain injury is a known risk factor for lung damage. First, swallowing disorders are frequent in brain-injured patients up to the fact that aspiration pneumonia causes the highest attributable mortality of all medical complications following stroke [26, 27]. Aspiration of gastric contents is consistently associated with severe aspiration pneumonia, and it also contributes to the development of ALI/ARDS, as reported in previous studies [28]. Our study also found that patients with vomiting or aspiration were more likely to develop ALI/ARDS. Junsong Wu et al. found that the occurrence of chest trauma, aspiration and vomiting in severe craniocerebral injury patient had a direct impact on the development of ARDS [29]. We hypothesize that these patients tend to develop aspiration pneumonia, which increases the probability of pulmonary infections and inadequate ventilation, and promotes the development and progression of ALI/ARDS. Vomit aspiration causes acidic gastric juice to enter the trachea, leading to chemical lung injury and increased permeability due to endothelial damage of pulmonary capillaries, causing interstitial and alveolar edema, which is another cause of ALI/ARDS secondary to SAH patient. Therefore, more extensive measures probably are taken to prevent the aspiration of gastric contents.

Elderly patient has been demonstrated to be a risk factor for mortality from trauma in the past [30]. The differences in the incidence rates between men and women were not statistically significant. Further divided into different age groups, statistical analysis of the incidence of ALI/ARDS in different age groups showed that the incidence rates of < 60 and ≥ 60 years old groups were 17.3% and 36.0%, respectively, with statistically significant differences in incidence rates (*p* < 0.05). This indicates that elderly patients are more likely to develop ALI/ARDS, which may be related to more coexisting diseases, lower organ function, and more pronounced systemic inflammatory response in elderly patients. Additionally, the effect of age on the development of ALI/ARDS may be modified by disease-specific characteristics, and the higher age-specific incidence of SAH in older cohorts [31].

We performed a logistic multi-factor regression analysis, and the results showed that GCS, SAPS II and Hunt-Hess classification were independent risk factors predicting the appearance of ALI/ARDS. It was indicated that the clinical grade of hemorrhage persisted as a strong independent predictor of the development of ALI/ARDS. Therefore, when treating SAH early in clinical practice, special attention should be paid to patients with SAH with higher SAPS II and Hunt-Hess grades, lower GCS grade. While actively managing intracranial vascular disease, dynamic monitoring of the lungs and other extracranial vital organs should be emphasized to achieve early detection of ALI/ARDS and take active and effective preventive treatment measures to reduce incidence and improve the cure rate and patient's survival quality. Previous studies showed that respiratory disease was associated with an increased odds of developing ARDS after TBI [32]. However, no relationship between ARDS and SAH was observed in our study. On the other hand, patients with aspiration or aspiration pneumonia are often more likely to develop hypoxemia and respiratory distress [33], eventually leading to the development of ALI/ARDS.

Our study demonstrated that ALI/ARDS occurring after SAH is associated with increased poor prognosis, tracheostomy, hospital complications and prolonged ICU days, ventilator days and hospital stay. Our observation is in line with the other study, and ALI/ARDS significantly increases adverse outcome and mortality in SAH patients [5]. Although no deaths were reported in our study, patients with ALI/ARDS are indeed more likely to exhibit a poor neurological prognosis. The pathophysiological mechanisms by which patients with SAH in combination with ALI/ARDS exhibit a worse neurological prognosis may be multifaceted. Inadequate cerebral oxygen supply transmitted to cells leading to neuronal hypoxia and neuronal death is one of the main hypotheses. As those results suggesting that recognition and treatment of ALI/ARDS in patients with SAH could possibly improve clinical outcome. We will then continue to expand the cohort size and extend the follow-up period to observe the effect of ALI/ARDS on mortality in patients with SAH.

Our study has several limitations. Our cohort was composed of patients from a single medical institution, and therefore our results may not easily generalize. It is may not be feasible to promote our findings in other clinical settings. However, the risk factors and prognosis in this study are comparable to other large multicenter reports [5, 20, 33], making the cohort somewhat representative. Furthermore, the sample size of our study was small and the follow-up period was not long enough to have long-term prognostic information of the patients.

## Conclusions

The results of this study indicate that the development of ALI/ARDS is a severe complication of aSAH, and increasing age, lower GCS, higher Hunt-Hess grade, higher SAPS II score, gastric aspiration or pneumonia, hypoxemia, and tachypnea are risk factors for ALI/ARDS. In addition, ALI/ARDS patients exhibited worse GOS scores, higher tracheotomy and hospital complication rates. And has a longer duration of mechanical ventilation, ICU length and hospitalization of stay. Patients with these identified risk factors require aggressive treatment as early as possible and thereby improve the clinical prognosis.

## Data Availability

The data of this study are not openly available due to the sensitive nature of these data and are available from the corresponding author upon reasonable request.
